# Molecular subtype identification of cerebral ischemic stroke based on ferroptosis-related genes

**DOI:** 10.1038/s41598-024-53327-2

**Published:** 2024-04-23

**Authors:** Yufeng Wang, Xinjuan Xu, Xinjun Shui, Ruilin Ren, Yu Liu

**Affiliations:** 1 Department of Neurosurgery, Shanxi Cardiovascular Hospital, No.18, Yifen Street, Taiyuan City, 030024 Shanxi Province China; 2 Department of Surgical, Peking University First Hospital Taiyuan, Taiyuan, China

**Keywords:** Genomic analysis, Genetics research

## Abstract

Cerebral ischemic stroke (CIS) has the characteristics of a high incidence, disability, and mortality rate. Here, we aimed to explore the potential pathogenic mechanisms of ferroptosis-related genes (FRGs) in CIS. Three microarray datasets from the Gene Expression Omnibus (GEO) database were utilized to analyze differentially expressed genes (DEGs) between CIS and normal controls. FRGs were obtained from a literature report and the FerrDb database. Weighted gene co-expression network analysis (WGCNA) and protein–protein interaction (PPI) network were used to screen hub genes. The receiver operating characteristic (ROC) curve was adopted to evaluate the diagnostic value of key genes in CIS, followed by analysis of immune microenvironment, transcription factor (TF) regulatory network, drug prediction, and molecular docking. In total, 128 CIS samples were divided into 2 subgroups after clustering analysis. Compared with cluster A, 1560 DEGs were identified in cluster B. After the construction of the WGCNA and PPI network, 5 hub genes, including MAPK3, WAS, DNAJC5, PRKCD, and GRB2, were identified for CIS. Interestingly, MAPK3 was a FRG that differentially expressed between cluster A and cluster B. The expression levels of 5 hub genes were all specifically highly in cluster A subtype. It is noted that neutrophils were the most positively correlated with all 5 real hub genes. PRKCD was one of the target genes of FASUDIL. In conclusion, five real hub genes were identified as potential diagnostic markers, which can distinguish the two subtypes well.

## Introduction

Cerebral ischemic stroke (CIS) is a symptom of the focal or complete neurological deficit caused by cerebral blood supply dysfunction, hypoxia and ischemia. CIS has the characteristics of a high incidence and mortality rate, which endangers human physical and mental health^1,2^. The lack of blood supply and reperfusion lead to the interruption of energy and redox homeostasis in the cerebra, caused the metabolic disorder of lipid oxides in cells, and then leads to abnormal metabolism under the catalysis of iron-dependent phospholipid peroxidation. The aggregation of these products destroys the redox balance and the homeostasis of iron metabolism in cells, triggering regulatory cell necrosis, which is called ferroptosis^3,4^. Ferroptosis is significantly different from apoptosis and cell necrosis at the morphological, biological, and gene levels^5^. In recent years, a series of research reports demonstrated that ferroptosis is closely related to CIS^6–8^.

Ferroptosis is caused by a redox imbalance between the production of oxidants and antioxidants, which can be precisely regulated at multiple levels^9^. Increasing evidence supported ferroptosis as a mechanism of acute ischemic stroke in vivo^10^. During the cerebral ischemic episode, iron overload is found in the damaged area of the cerebra^11,12^. The imbalance of iron metabolism, including its overload and deficiency, has been shown to impact ischemic stroke outcomes^13^. The regulation of ferroptosis can intervene the process of stroke in animal models, suggesting that ferroptosis is a novel potential target for the treatment of stroke^14^. In addition, ferroptosis-related gene (FRG) signature had been reported to have the potential as ischemic stroke diagnostic markers^15^.

In weighted gene co-expression network analysis (WGCNA), the genes with similar expression patterns were clustered into a module, and the correlation between the module and phenotype was analyzed^16,17^. WGCNA can be used to find genes with key functions, help identify potential mechanisms, and explore candidate biomarkers^18,19^. Herein, we first clustered samples based on expression patterns of FRGs to obtain different subtypes of CIS. Based on the differential expressed genes (DEGs) between different subtypes, we used WGCNA to screen the key modules of diseases and get the hub genes related to ferroptosis. Later, we also constructed a multi-factor regulatory network for the screened key hub genes to analyze their expression in CIS and subclusters.

## Methods

### Datasets acquisition and preprocessing

We used the keywords "stroke" and "homo sapiens" to filter the gene expression profile data in the Gene Expression Omnibus (GEO) database. Then the corresponding dataset was screened using the following criteria: (1) sample size is ≥ 5; (2) normal controls were included in the dataset. The exclusion criteria were as follows: (1) studies performed at the cell line or animal level; (2) single cohort study; (3) repeated or overlapping studies. Three datasets of CIS, GSE16561, GSE22255, and GSE58294, were included in our study. The GSE16561 includes peripheral blood samples of 39 CIS patients and 24 healthy controls, GSE22255 includes peripheral blood samples of 20 CIS patients and 20 normal controls, and GSE58294 includes peripheral blood samples of 69 patients with CIS and 23 normal controls. After downloading the submitted gene expression matrix file, the gene expression profile were annotate using the GPL platform annotation file, and the gene probes were convert into gene symbols. The combat function in the R package "SVA"^20^ was used to remove batch effects.

### Acquisition of FRGs

The list of FRGs were obtained from the FerrDb (http://www.zhounan.org/ferrdb/index.html) database, a database dedicates to ferroptosis regulators and ferroptosis-disease associations^21^, supplemented by a previous literature^22^. A total of 267 FRGs were obtained finally. Among which, 216 FRGs were detected in three CIS datasets.

### Identification of DEGs among different groups

After data preprocessing of the above three datasets, the "limma" (3.36.5) package was used to obtain the DEGs between CIS and normal controls^23^. The screened standard was false discovery rate (FDR) < 0.05 and |log_2_ fold change (FC)| > 0.2^24^. Differentially expressed FRGs were further screened and displayed in the heat map. Samples were grouped using the "ConsensusClusterPlus" (1.44.0) package in R^25^, and the parameters were set as maxK = 5, reps = 1000, pItem = 0.8, pFeature = 1, clusterAlg = "pam". The optimal k was determined according to cumulative distribution function (CDF) and area under CDF curve. The DEGs among different CIS subtypes were screened based on FDR < 0.05 and |log_2_FC| > 0.3. The principal component analysis (PCA) using the prcomp function of R^26^ was performed to evaluate the accuracy of grouping.

### Functional analysis and gene set enrichment analysis (GSEA)

To analyze the DEGs involved in biochemical processes and pathways in the development of CIS, the kyoto encyclopedia of genes and genomes (KEGG) pathway enrichment^27,28^ and gene ontology (GO) analysis were performed using Database for Annotation, Visualization, and Integrated Discovery (DAVID, https://david.ncifcrf.gov/)^29,30^ with p < 0.05. Top 20 enrichment results were visualized by the "GOplot" of R. In addition, GSEA was conducted to find out enriched gene sets with hallmark gene sets (h.all.v2022.1.Hs.symbols.gmt) downloaded from Molecular Signatures Database (MSigDB). Enrichment results with FDR < 0.05 were considered statistically significant.

### WGCNA

The R-package "WGCNA" (1.67)^31,32^ was used to construct a scale-free gene co-expression network. The "hclust" function was utilized to cluster the sample data to delete outliers. The "pickSoftThreshold" function was used to calculate an appropriate soft threshold power. The height was set to 0.90. Then, the adjacency matrix was calculated and transformed into the topological overlap matrix (TOM) and the corresponding dissimilarity matrix (1 − TOM). Genes with similar expression patterns were clustered together and divided into modules with default parameters according to the "cutreeDynamic" function. The identified modules were truncated and merged with a height of 0.25. The model characteristic gene (ME) is defined as the first principal component in each module, which can summarize the expression pattern of all genes in the module. To determine the key modules most related to CIS, the "moduleEigengenes" function was applied to calculate the ME of each module, and the correlation between ME and CIS was analyzed by the "Pearson" method. The module with the highest correlation with CIS was selected as the hub module. The candidate hub genes were selected based on the module connectivity and clinical trait relationship. The gene significance (GS) and module membership (MM) were calculated to select candidate hub genes with GS > 0.5 and MM > 0.8.

### Screening hub genes

All genes in the hub module were imported into the STRING (https://cn.string-db.org/) database^33^ to build the protein–protein interaction (PPI) network and visualized by Cytoscape (3.9.0)^34^. The CytoHubba, plug-in in Cytoscape, was used to screen the central genes in the PPI network (gene of the top 40-degree). By overlapping the candidate hub genes in the hub module and the central genes in the PPI network, the real hub genes were obtained. The Pearson correlation among the real hub genes was calculated and the expression of the real hub genes between two groups was pairwise compared. The receiver operating characteristic (ROC) curve analysis was adopted to evaluate the accuracy of the real hub genes in distinguishing different CIS subtypes, and normal controls and CIS patients.

### Immune microenvironment analysis

CIBERSORT^35^ was used to calculate the proportion of 22 immune cells in each sample based on gene expression patterns. The difference in immune cells among different subgroups were calculated and visualized using a bar plot. The percentage of different immune cells in each sample was shown in the column accumulation chart. In addition, Pearson correlation coefficient method was used to calculate the correlation between real hub genes and differential immune cells.

### Construction of transcription factor (TF) regulatory network, drug prediction and molecular docking

In order to explore the effect of TFs regulating gene expression on the occurrence and development of CIS, the regulation network of TF-hub gene was built based on TRRUST (https://www.grnpedia.org/trrust/) database^36^. In addition, real hub gene related drugs were identified from the DGIdb (https://dgidb.org/) database^37^, followed by molecular docking by AutoDock Vina 1.1.2 software^38^. The docking results were visualized through PyMol 2.5^39^.

### Statistical analysis

R software (v3.5.3) was used for all statistical analysis. The "limma" (3.36.5) package was used to obtain the DEGs. Consensus clustering analysis was performed using the "ConsensusClusterPlus" (1.44.0) package in R. The PCA using the prcomp function of R was performed to evaluate the accuracy of grouping. The R-package "WGCNA" (1.67) was used to construct a scale-free gene co-expression network and select candidate hub genes with GS > 0.5 and MM > 0.8. CIBERSORT was used to perform immune infiltration analysis. Unless otherwise stated, a p-value < 0.05 was deemed statistically significant.

## Results

### DEGs in the CIS samples

**Figure 1:**
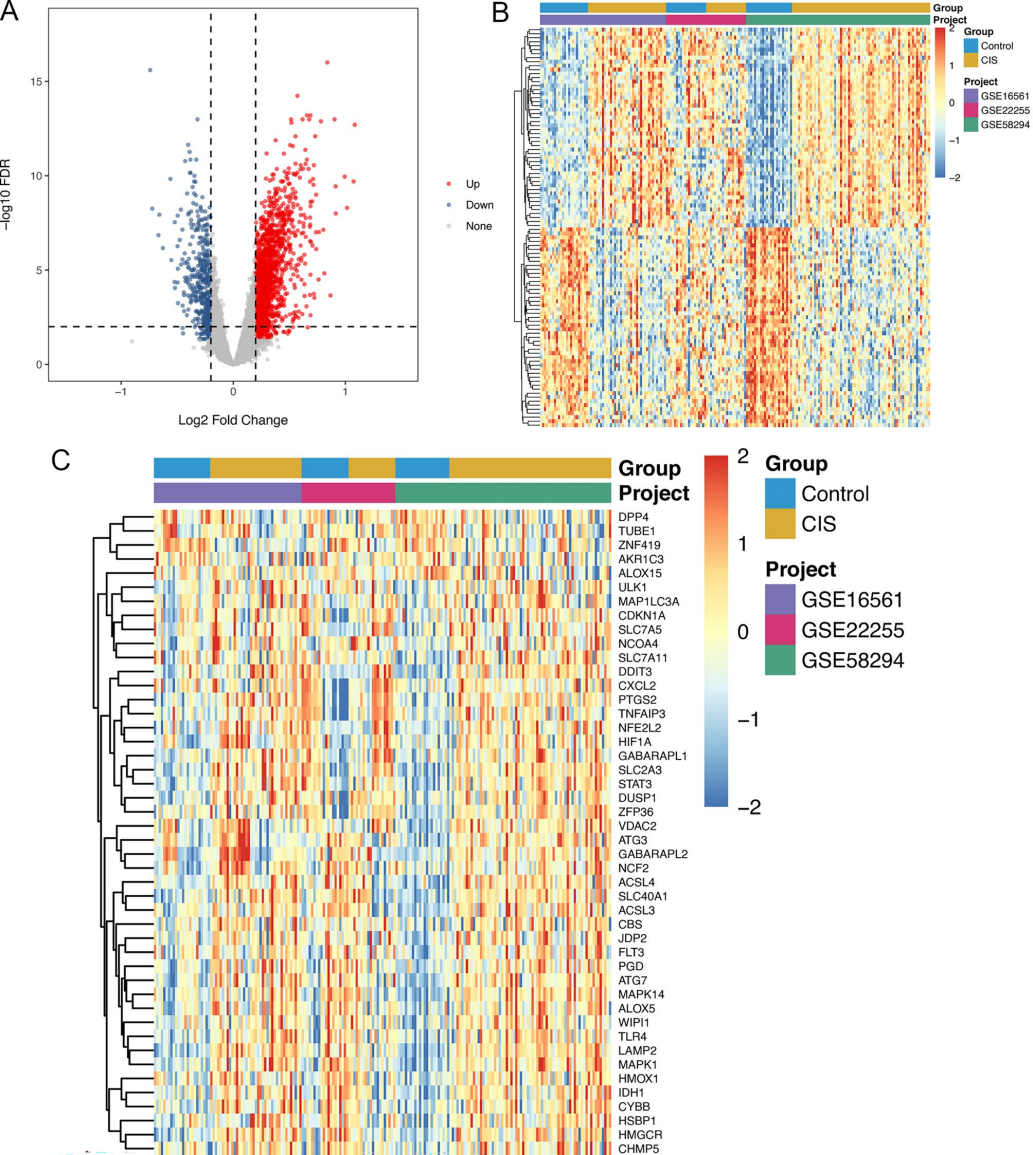
DEGs between CIS patients and healthy controls. (**A**) volcanic map of 1995 DEGs between CIS group and healthy controls; (**B**) heat map of 1995 DEGs; (**C**) heat map of 46 differentially expressed FRGs between CIS group and health controls.

The combined dataset included 67 healthy controls and 128 CIS samples. A total of 13,521 genes in the three datasets were intersected after removing the batch effect. A total of 1995 DEGs were identified in the CIS group, of which 1518 genes were up-regulated, and 477 genes were down-regulated (Fig. 1A,B). Totally, 46 differentially expressed FRGs were identified, of which 41 were up-regulated and 5 were down-regulated (Fig. 1C).

### DEGs between two CIS subtypes

**Figure 2:**
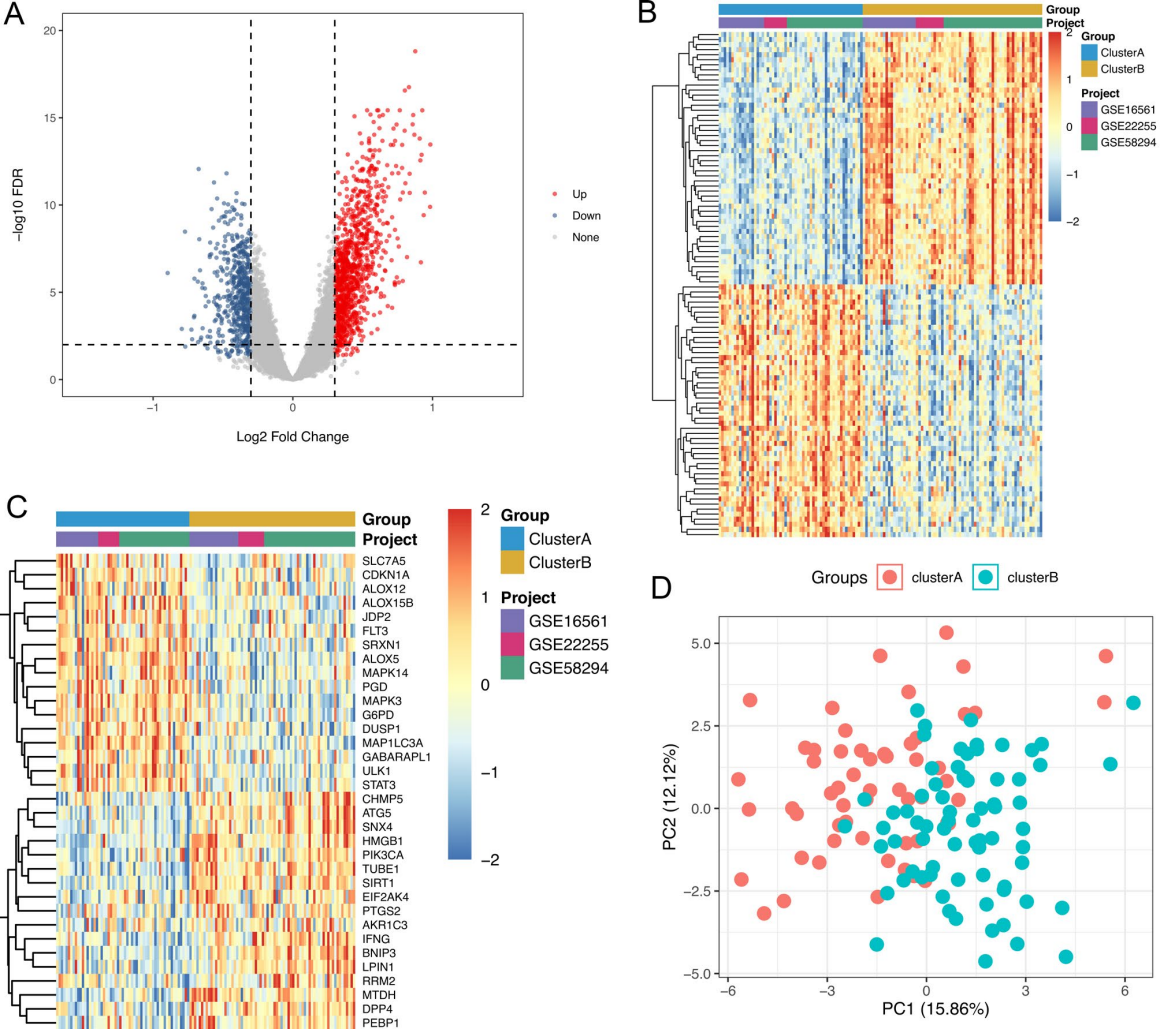
DEGs between cluster A and B in CIS patients. (**A**) volcanic map of 1560 DEGs between cluster A and cluster B in CIS patients; (**B**) heat map of 1560 DEGs between cluster A and cluster B; (**C**) heat map of 34 differentially expressed FRGs between cluster A and cluster B; (**D**) Results from PCA for cluster A and cluster B in CIS patients.

**Figure 3:**
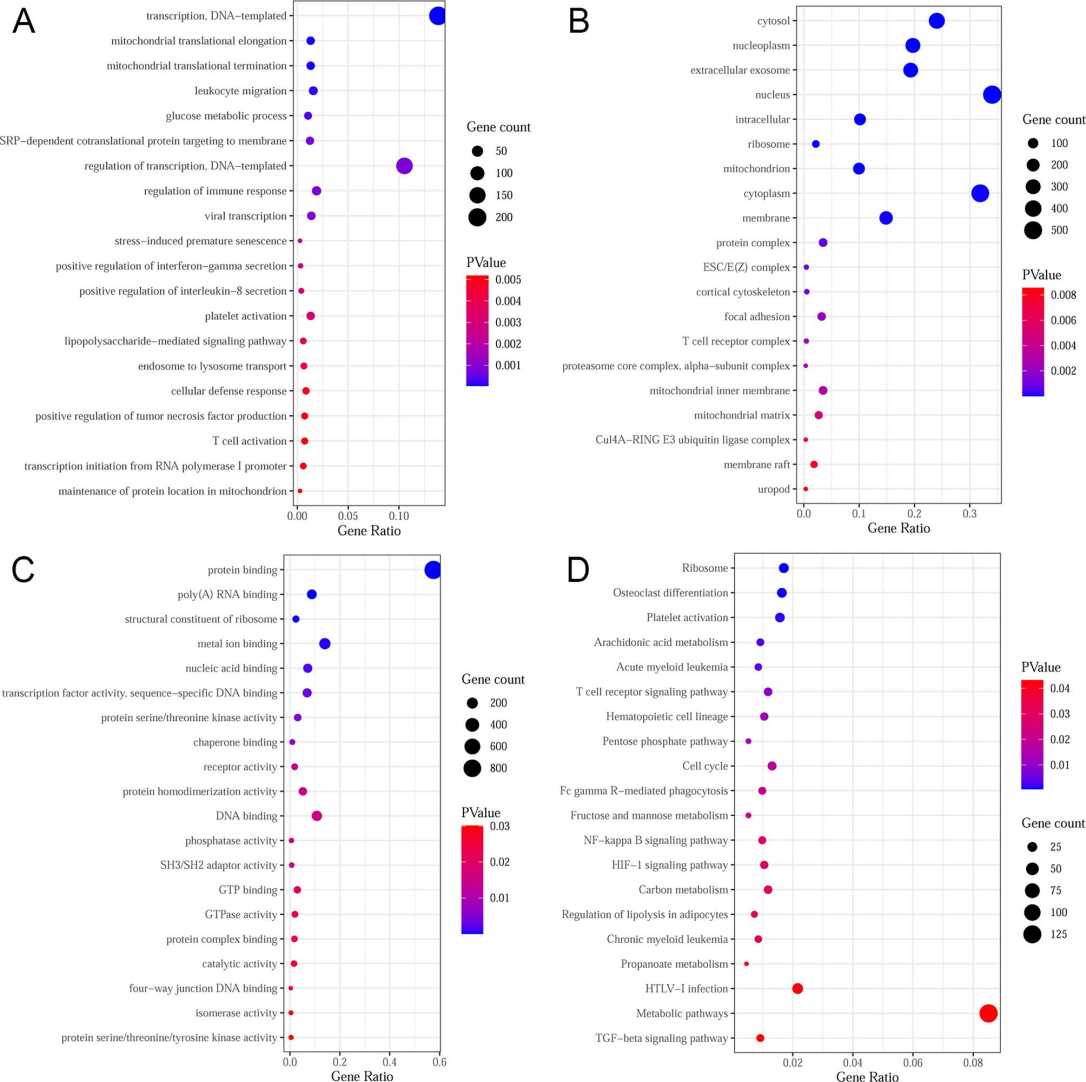
Top 20 enrichment results of DEGs between cluster A and cluster B in CIS patients. (**A**) BP; (**B**) CC; (**C**) MF; (**D**) KEGG.

According to the CDF value and CDF area change, k = 2 exhibited the best unsupervised clustering competence (Fig. S1). A total of 128 CIS samples were divided into 2 subgroups after cluster analysis: cluster A and B (Table 1). Compared with cluster A, 1560 DEGs were identified in cluster B (1004 up-regulated and 556 down-regulated, Fig. 2A,B). Among which, 34 differentially expressed FRGs were screened, of which 17 genes were up-regulated and 17 genes were down-regulated (Fig. 2C). Result of PCA was displayed in Fig. 2D. GO enrichment results showed that most of the DEGs were distributed in cytosol, nucleoplasm, extracellular exosome, and nucleus, and involved in regulation of transcription and immune response, protein binding, and other biological processes (Fig. 3A–C). KEGG enrichment analysis showed that DEGs were enriched in NF-kappa B signaling pathway, HIF-1 signaling pathway, TGF-beta signaling pathway, and metabolic pathways (Fig. 3D). GSEA results indicated that the pathways of MYOGENESIS, COAGULATION, APICAL JUNCTION, HEME METABOLISM, HEDGEHOG SIGNALING, KRAS SIGNALING DN, REACTIVE OXYGEN SPECIES PATHWAY, P53 PATHWAY, HYPOXIA, UV RESPONSE UP, INFLAMMATORY RESPONSE, and XENOBIOTIC METABOLISM were more active in cluster A, while MYC TARGETS V1, E2F TARGETS, OXIDATIVE PHOSPHORYLATION, G2M CHECKPOINT, UNFOLDED PROTEIN RESPONSE, MTORC1 SIGNALING, ANDROGEN RESPONSE, DNA REPAIR, PROTEIN SECRETION, and MYC TARGETS V2 were more active in cluster B. The pathways with FDR < 0.01 were displayed in Fig. S2.

**Table 1:** The consistent clustering grouping of CIS samples.

GEO ID	Cluster A	Cluster B
GSE16561	18	21
GSE22255	9	11
GSE58294	30	39
Total	57	71

### Construction of WGCNA

**Figure 4:**
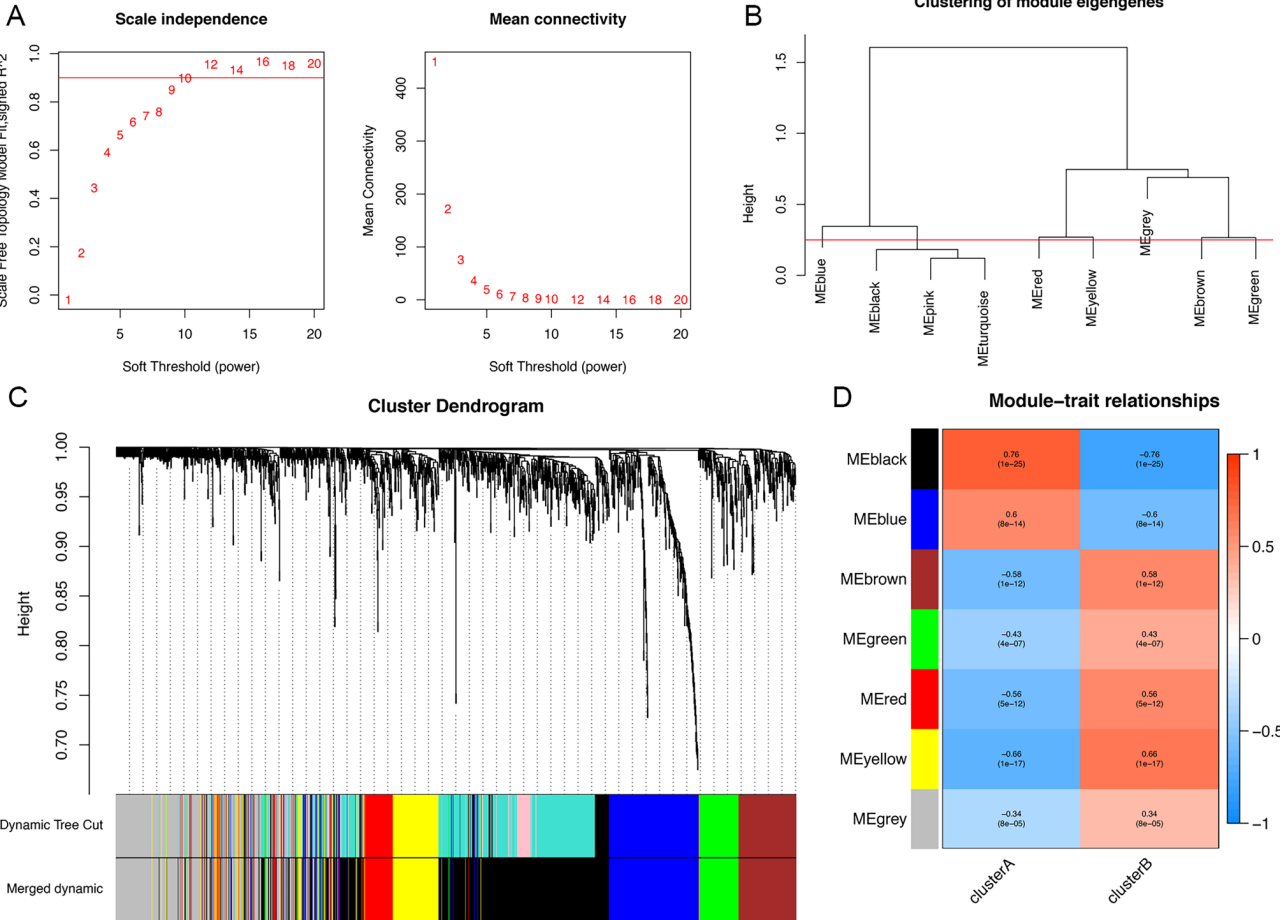
Construction of WGCNA. (**A**) Determination of soft thresholding power in the WGCNA; (**B**) the merged modules for clustering by WGCNA; (**C**) clustering dendrogram of genes, and different colors represent different modules; (**D**) the heat map of module-trait relationship in CIS, the black module has the highest correlation with CIS.

WGCNA was utilized to analyze 1560 DEGs from 128 samples to identify CIS-related genes. The power β = 10 was selected to ensure a scale-free topology (Fig. 4A). Then, 6 modules were determined (Fig. 4B,C). Among the six modules, the black module had the highest correlation with CIS with Pearson r = 0.76 (P = 1e−25, Fig. 4D). Therefore, the black module was considered the hub module, which including 508 genes.

### Identification of the real hub genes

**Figure 5:**
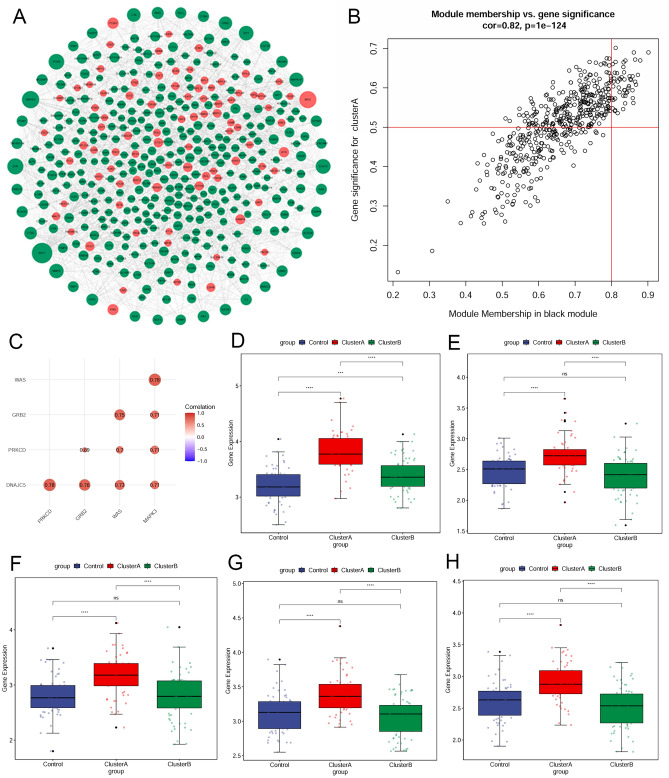
Identification of the real hub genes. (**A**) PPI network of 508 genes in the black module; (**B**) Scatterplot of genes (module membership versus gene significance) in the black module; (**C**) Correlation analysis among the five real hub genes; (**D**–**H**) The expression levels of five genes in normal controls, cluster A, and cluster B. (**D**) WAS; (**E**) DNAJC5; (**F**) PRKCD; (**G**) GRB2; (**H**) MAPK3.

A total of 508 genes in the black module were imported into the STRING database to construct a PPI network, which includes 429 nodes and 1674 edges (Fig. 5A), and the top 40 ranked degree was defined as the central node genes. Fifty-two genes were selected as candidate hub genes in the black module according to GS > 0.5 and MM > 0.8 (Fig. 5B). By overlapping the candidate hub genes and the central node genes, five intersection genes were considered as the real hub genes, including MAPK3, WAS, DNAJC5, PRKCD, and GRB2. The correlation analysis showed that there were strong correlations among the five real hub genes (Fig. 5C). MAPK3 had a strong correlation with the other 4 hub genes, especially with WAS (coefficient = 0.78). Among which, MAPK3 was a FRG differentially expressed between cluster A and cluster B. These 5 real hub genes all exhibited specifically highly expressed in cluster A (Fig. 5D–H).

### ROC analysis of real hub genes

**Figure 6:**
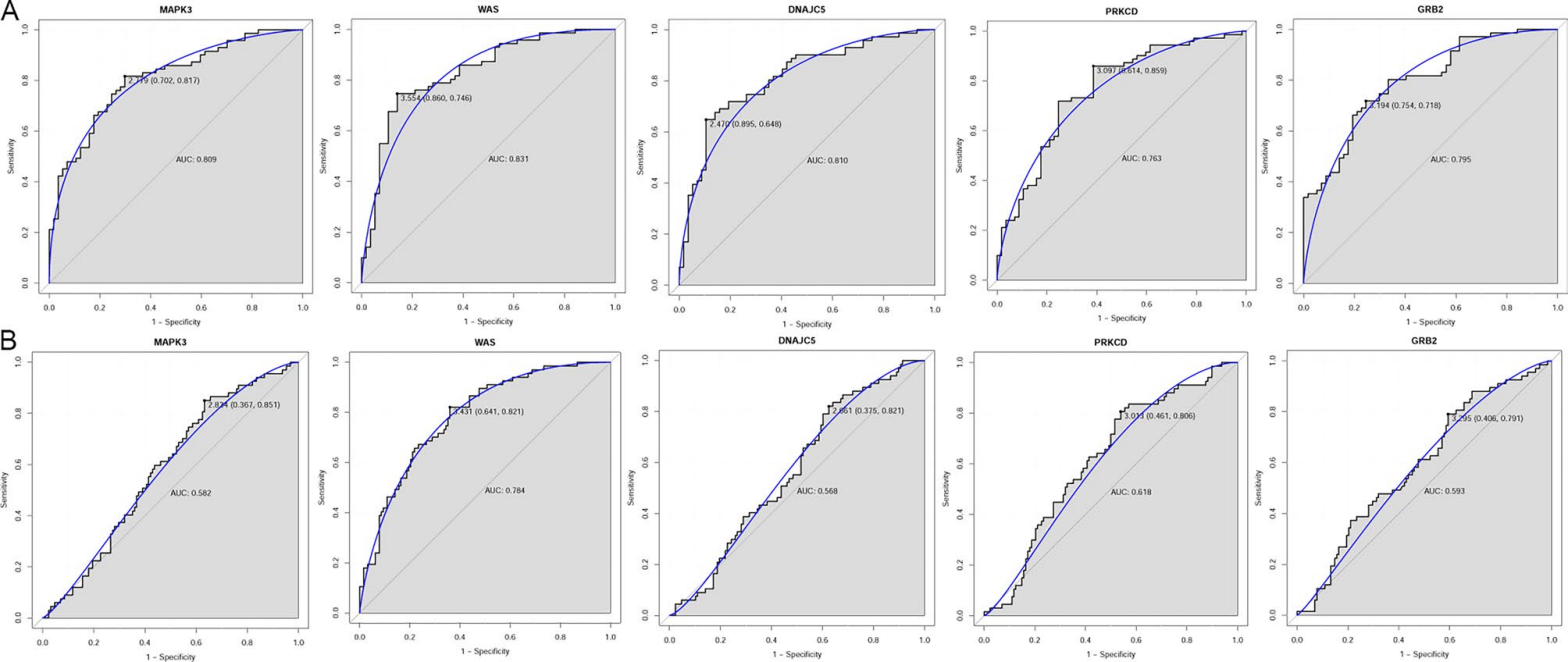
The ROC curve of key genes in CIS patients. (**A**) ROC analysis of MAPK3, WAS, DNAJC5, PRKCD, and GRB2 between cluster A and cluster B; (**B**) ROC analysis of MAPK3, WAS, DNAJC5, PRKCD, and GRB2 between CIS patients and normal control.

To further evaluate the accuracy of these 5 hub genes in distinguishing cluster A and cluster B in patients with CIS, ROC of the 5 hub genes were performed. The results (Fig. 6A) showed that the AUC values of all 5 genes were relatively high, showing good sensitivity and specificity. We also evaluated the diagnostic values of 5 genes in distinguishing CIS patients from normal controls (Fig. 6B). The results demonstrate that the AUC value of WAS gene was relatively high, with good sensitivity and specificity.

### Immune microenvironment analysis

**Figure 7:**
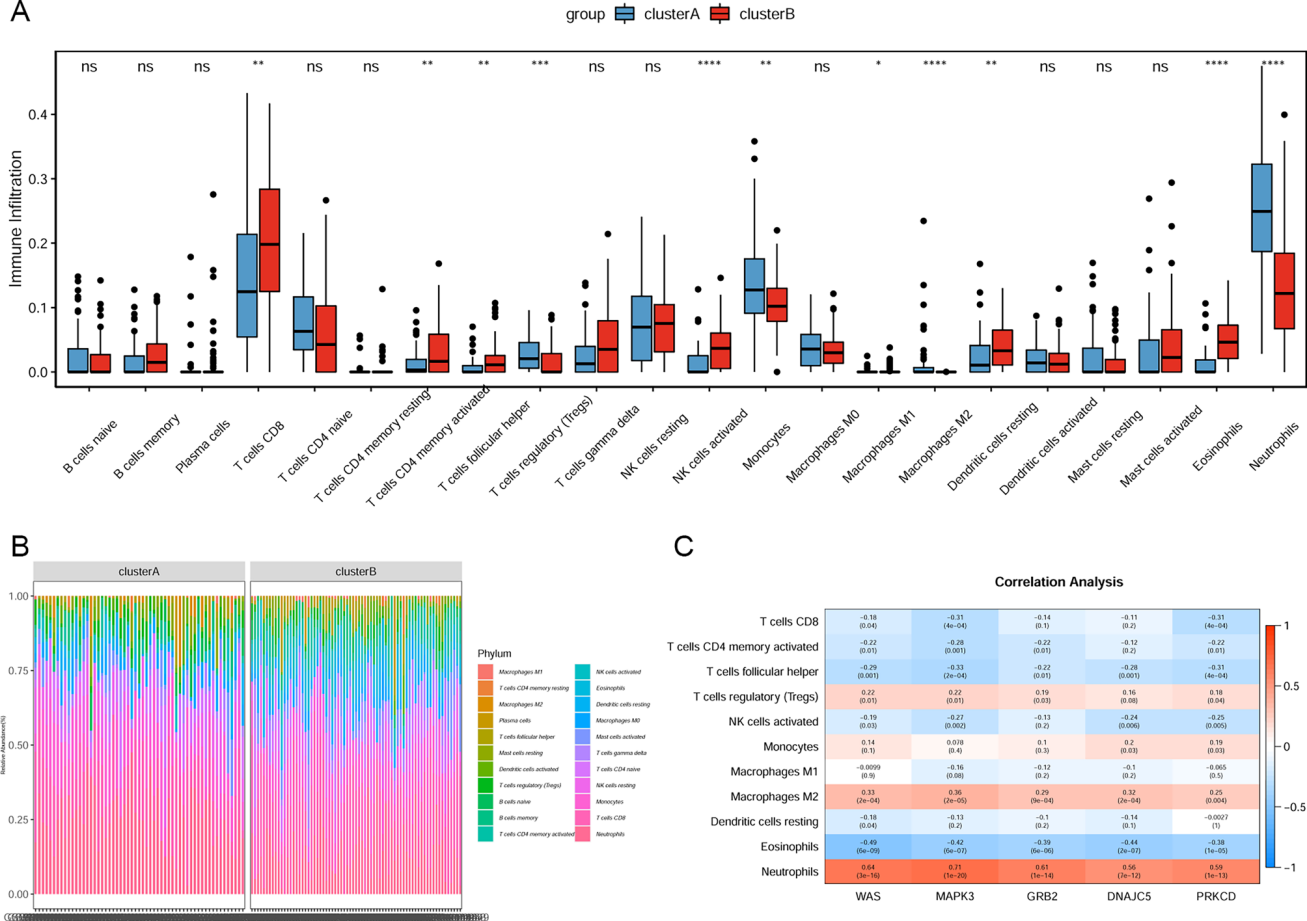
Immune microenvironment analysis between cluster A subtype and cluster B subtypes. (**A**) boxplot of immune infiltration of 22 immune cells; (**B**) the heat map of relative abundance of 22 immune cells; (**C**) pearson correlation analysis between immune cells and 5 real hub genes.

Based on the immune microenvironment analysis, the immune infiltration levels of NK cells activated and Eosinophils were the most significantly increased, and Macrophages M2 and Neutrophils were the most significantly decreased in cluster B subtype, compared with cluster A subtype (Fig. 7A). The heat map of relative abundance of 22 immune cells is shown in Fig. 7B. It is noted that neutrophils were the most positively correlated with all 5 real hub genes (Fig. 7C).

### TF regulatory network, drug prediction and molecular docking of 5 real hub genes

**Figure 8:**
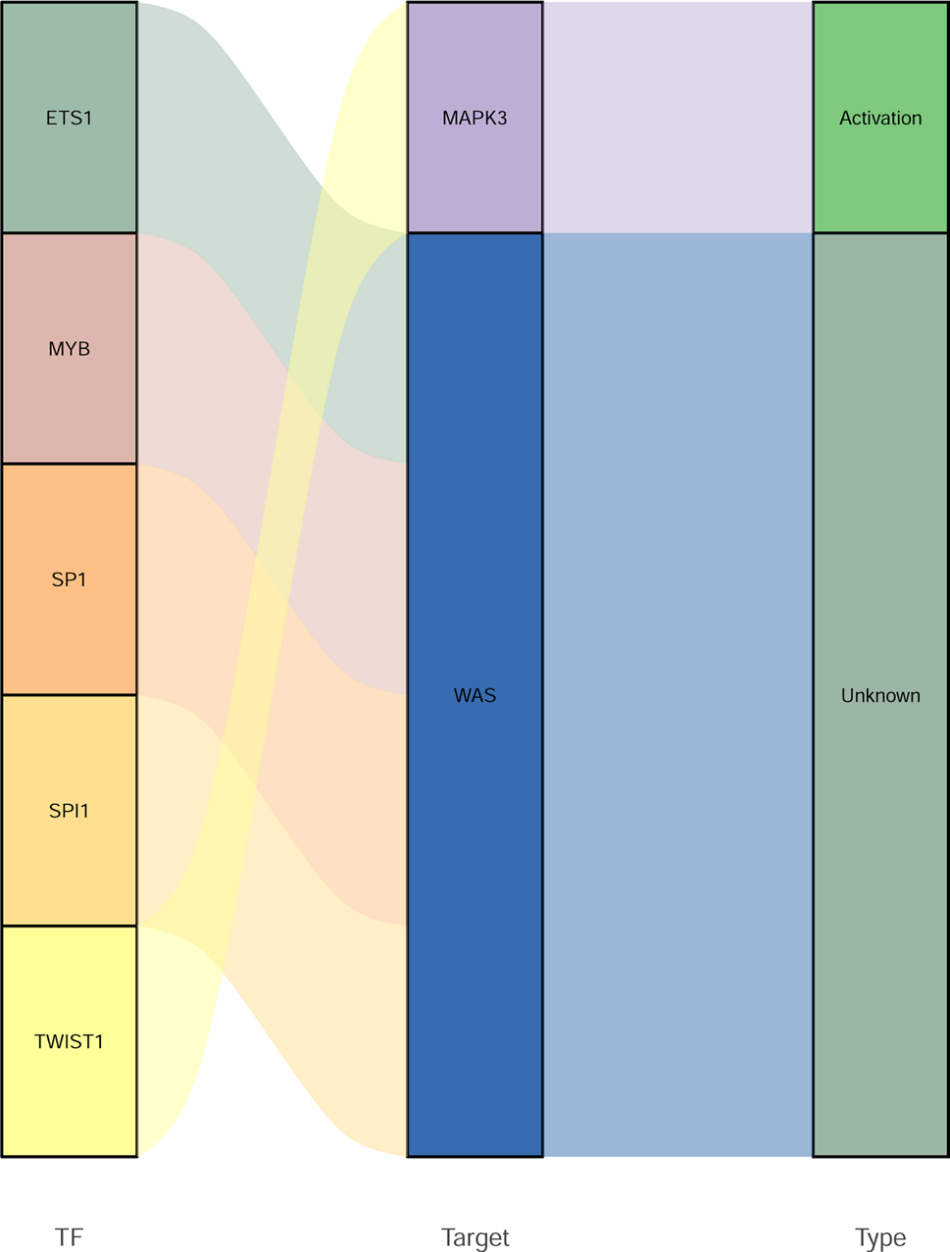
The regulatory network between TFs and 5 real hub genes.

**Figure 9:**
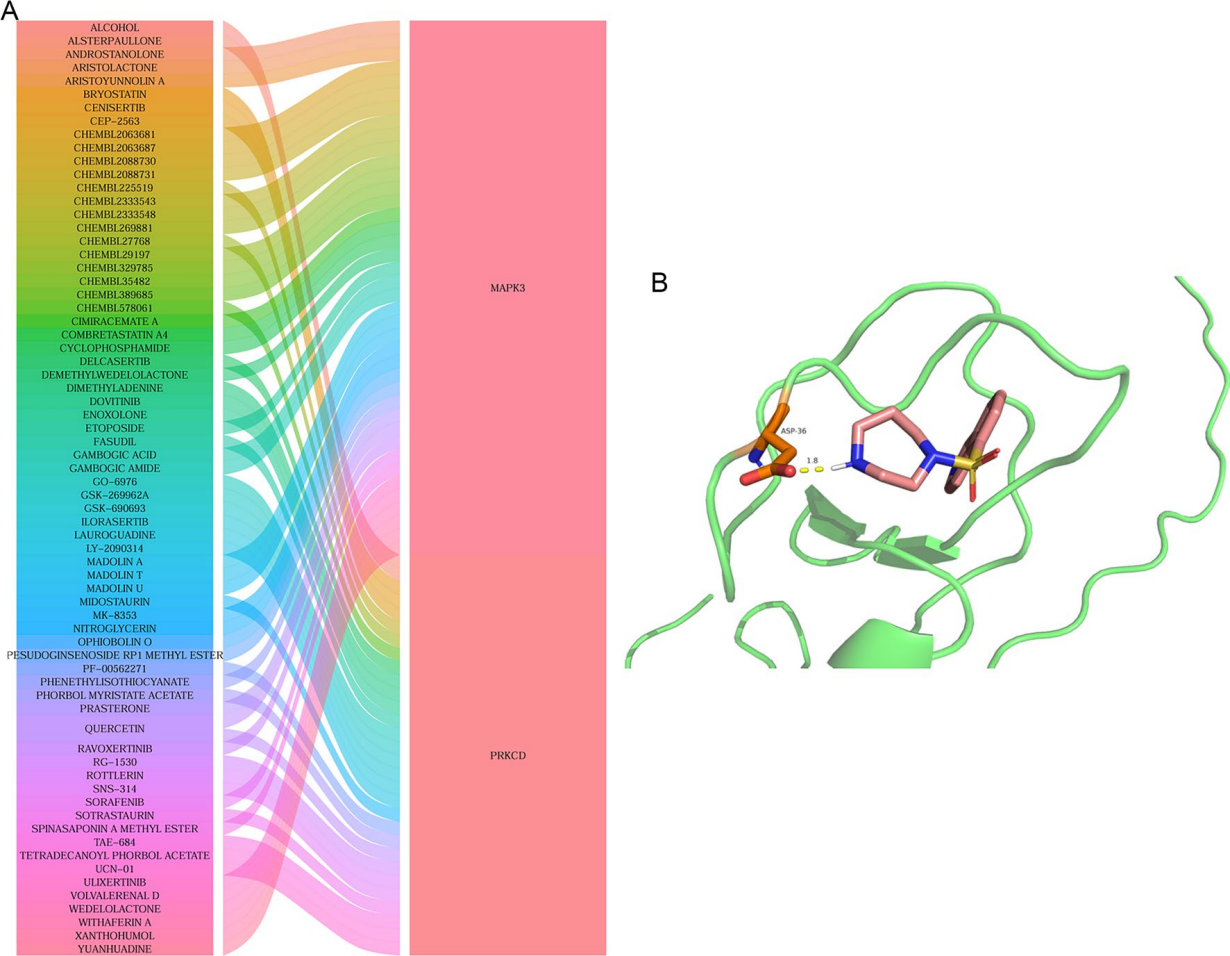
Drug prediction and molecular docking. (**A**) drug prediction of 5 real hub genes based on the DGIdb database; (**B**) molecular docking of FASUDIL-PRKCD.

Based on TRRUST database, only two hub genes, MAPK3 and WAS, related TFs were identified. Twist family bHLH transcription factor 1 (TWIST1) regulated MAPK3. ETS proto-oncogene 1, transcription factor (ETS1), MYB proto-oncogene, transcription factor (MYB), Sp1 transcription factor (SP1), and Spi-1 proto-oncogene (SPI1) regulated WAS (Fig. 8). In addition, 5 real hub genes related drugs were queried based on the DGIdb database. The result showed that only MAPK3 and PRKCD were found to be targeted by multiple drugs. For example, PRKCD was one of the target gene of FASUDIL (Fig. 9A). The binding energy of PRKCD and FASUDIL was − 7.06 kcal/mol, forming a hydrogen bond with ASP-36 residues (Fig. 9B).

## Discussion

CIS refers to the brain tissue necrosis caused by stenosis or occlusion of cerebral blood supply arteries (carotid artery and vertebral artery) and insufficient cerebral blood supply. The pathogenesis of ischemic stroke is complex. At present, reported pathological mechanisms of CIS include abnormal energy metabolism, excitotoxicity of amino acids, oxidative damage of free radicals and apoptosis^40–44^. At the cellular level, hypoperfusion, hypoxia, and glucose deficiency cause insufficient energy production in brain metabolism, thus leading to some toxic metabolites accumulating locally, such as excitatory toxic products, acidic metabolites, oxidative stress products, and inflammatory mediators, resulting in cerebral cell dysfunction or death^45^. Various pathophysiological mechanisms had been reported to be involved in stroke, including angiogenesis, oxidative stress, autophagy formation, inflammatory response, and apoptosis^46^.

Inflammation is an important part of the pathogenesis of CIS. Dead or damaged cells release damage-associated molecular patterns in trauma (DAMP), thus leading to activated microglia, morphological changes occurring and secreting cytokines. Followed by astrocytes secreting cytokines and chemokines (including matrix metalloproteinases (MMPs) family expression), the chemokines participate in the immune response after injury, destructed the early blood–brain barrier after stroke. Further, peripheral lymphocytes and neutrophils transfer to the damaged brain area, and release proinflammatory cytokines, ROS and MMP, which aggravate brain parenchymal injury and blood–brain barrier damage^11^.

The pathological process of CIS is complex and involves mutiple signaling pathways. Sonic hedgehog (Shh) signaling pathway exerts neuroprotective effects in ischemic stroke^47^. A previous review summarized the roles of p53‑mediated ferroptosis signaling pathway underlying CIS^48^. It is reported that NF-kappa B could regulate microglial polarization after ischemic stroke^49^. The HIF-1 signaling pathway is activated under hypoxic conditions and plays important roles in the pathological processes of ischemic stroke^50^. In addition, it has been confirmed that activation of the HIF-1 signaling pathway is associated with ferroptosis^51^. It had been suggested that TGF-β1/Smad3 signaling pathway exhibited a protective role in CIS^52^. Functional enrichment analysis showed that DEGs between clusters A and B were enriched in NF-kappa B signaling pathway, HIF-1 signaling pathway, and TGF-beta signaling pathway. GSEA results indicated hedgehog signaling, p53 pathway, hypoxia, and inflammatory response were more active in cluster A.

As a “prototypic” or “classical” mitogen-activated protein (MAP) kinase, activation of MAPK3 protects cells from ischemic injury^53^. We found that MAPK3 was increased in cluster A compared to cluster B. Consistent with our study, Huang et al.^54^ found that MAPK3 was involved in the acute myocardial infarction process as a FRG. It is also necessary to analyze the correlation between this up-regulation and clinical classification in combination with the clinical characteristics of patients, and the degree of ischemia or nerve damage. On the other hand, the expression level of MAPK3 did not show difference between CIS patients and normal controls, which need to be further verified in the study of expanding the sample size and strictly controlling the control parameters. WAS is the coding gene of wiskott aldrich syndrome protein (WASP), which is involved in relaying signals from the surface of blood cells to the actin cytoskeleton^55^. Salvi and Thanabalu^56^ reported that expression of WASP was increased under hypoxic conditions. Our results also showed that WAS had the strongest correlation with MAPK3. However, the regulatory pathway and interaction of these two genes in CIS are still unclear, which needs further exploration and clarification.

DnaJ heat shock protein family (Hsp40) member C5 (DNAJC5) is a member of DNAJ family, and primarily expressed in neurons^57^. Currently, no report mentioned its roles in CIS, thus its roles and functions need further exploration. Protein Kinase C Delta (PRKCD) has been implicated in mediating ischemic and reperfusion damage in stroke-reperfusion^58^. Its upregulation is correlated with the occurrence of ischemic stroke. At present, several omics studies have shown that growth factor receptor-bound protein 2 (GRB2) is regulated by noncoding genes in ischemic stroke^59,60^, and its activation is critical for the activation of the Ras signaling pathway and downstream MAPK^61^. ROC analysis indicated that five real hub genes possessed potential diagnostic values, which can distinguish the two subtypes well. The association of hub genes with the disease characters, for example, age, sex, and duration of CIS even clinical classification, was not included in the analysis, which makes the clinical characteristics of cluster A and cluster B unclear. So it is needed to further establish a more reasonable patients’ cohort for biological information analysis.

Based on the immune microenvironment analysis, the infiltration levels of neutrophils were the most significantly decreased in cluster B subtype, compared with cluster A subtype. Moreover, neutrophils were the most positively correlated with MAPK3, WAS, DNAJC5, PRKCD, and GRB2. Infiltrating neutrophils increase blood–brain barrier permeability, which cause microvascular disorder. It is reported that neutrophil extracellular traps are significantly increased in the plasma of ischemic stroke patients and related to stroke severity and mortality^62^. Thus it can be seen that neutrophils play important roles in the development of CIS. Based on TRRUST database, MAPK3 and WAS related TFs were identified. TWIST1 regulated MAPK3. ETS1, MYB, SP1, and SPI1 regulated WAS. It is found that MACC1-AS1 exerts a protective role in hypoxia-induced brain microvascular endothelial cells in CIS via regulating miR-6867-5p/TWIST1^63^. CASC15 promotes cerebral ischemia/reperfusion injury via miR-338-3p/ETS1 axis in acute IS^64^. MiR-150 and its target MYB can form a negative feedback loop to control the level of post-stroke vascular endothelial growth factor (VEGF) expression^65^. In IS, miR-1224 contributes to dysfunction of natural killer cell by targeting Sp1 signaling^66^. In stroke, FTX transcript, XIST regulator (FTX) can regulate angiogenesis through miR-342-3p/SPI1 axis^67^. It is indicated that MAPK3 and WAS may be involved in the process of CIS under the regulation of TFs of TWIST1, ETS1, MYB, SP1, and SPI1. In addition, related drugs associated with MAPK3, WAS, DNAJC5, PRKCD, and GRB2 were queried based on the DGIdb database. The result showed PRKCD was one of the target gene of FASUDIL. Moreover, in the analysis of molecular docking, the binding score of PRKCD with FASUDIL was lower than − 6.0 kcal/mol, indicating a better binding affinity. It is suggested that PRKCD could be a drug target of FASUDIL in the treatment of CIS. It is noted that neutrophils was the most positively correlated with all 5 real hub genes. PRKCD was one of the target gene of FASUDIL.

In summary, our results showed that 5 key hub genes, MAPK3, WAS, DNAJC5, PRKCD, and GRB2, which were highly expressed in cluster A and can distinguish cluster A from cluster B of CIS. Among the 5 genes, MAPK3 was a FRG and WAS showed potential diagnostic value in distinguishing CIS patients from normal controls. In addition, we plan to collect a larger sample size for further validation of these five real hub genes identified in our study, followed by cell- and animal-level experiments to investigate their specific roles in CIS.

### Supplementary Information

Supplementary Legends.

Supplementary Figure S1.

Supplementary Figure S2.

## Data Availability

The datasets used and/or analysed during the current study are available from the corresponding author on reasonable request.
