# Supplementary material for: Molecular subtype identification of cerebral ischemic stroke based on ferroptosis-related genes
Source: Sci Rep. 2024 Apr 23;14:9350. doi: 10.1038/s41598-024-53327-2 (PMC11039763; doi:10.1038/s41598-024-53327-2)
Supplement: Supplementary file 1 — Supplementary Legends. [file 41598_2024_53327_MOESM1_ESM.docx]

**Supplementary figure legends**

**Figure S1** Consensus clustering.

**A**, Consensus clustering CDF for k = 2 to k = 5; **B**, Relative change in area under CDF curve for k = 2 to k = 5; **C**, Consensus clustering matrix for k = 2.

**Figure S2** The GSEA results showed up-regulated gene sets in cluster A (A) and cluster B (B) with FDR <0.01.
