# Supplementary figures and images for: Molecular subtype identification of cerebral ischemic stroke based on ferroptosis-related genes
Source: Sci Rep. 2024 Apr 23;14:9350. doi: 10.1038/s41598-024-53327-2 (PMC11039763; doi:10.1038/s41598-024-53327-2)

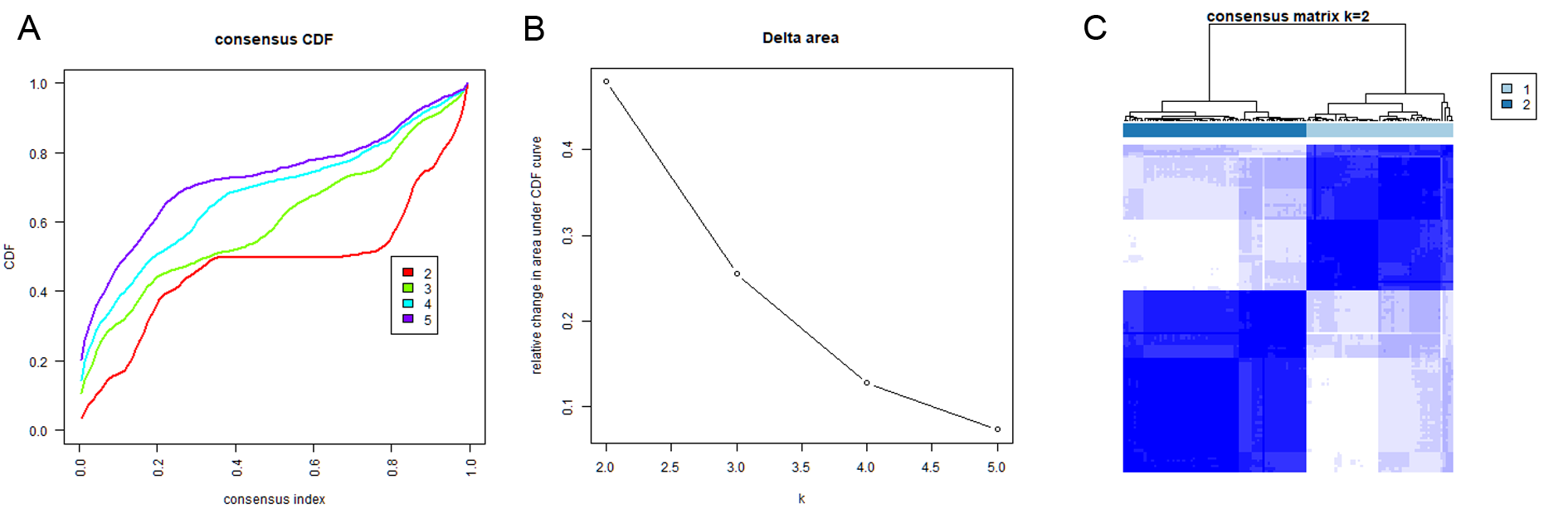

Supplement: Supplementary file 2 — Supplementary Figure S1. [file 41598_2024_53327_MOESM2_ESM.tif]

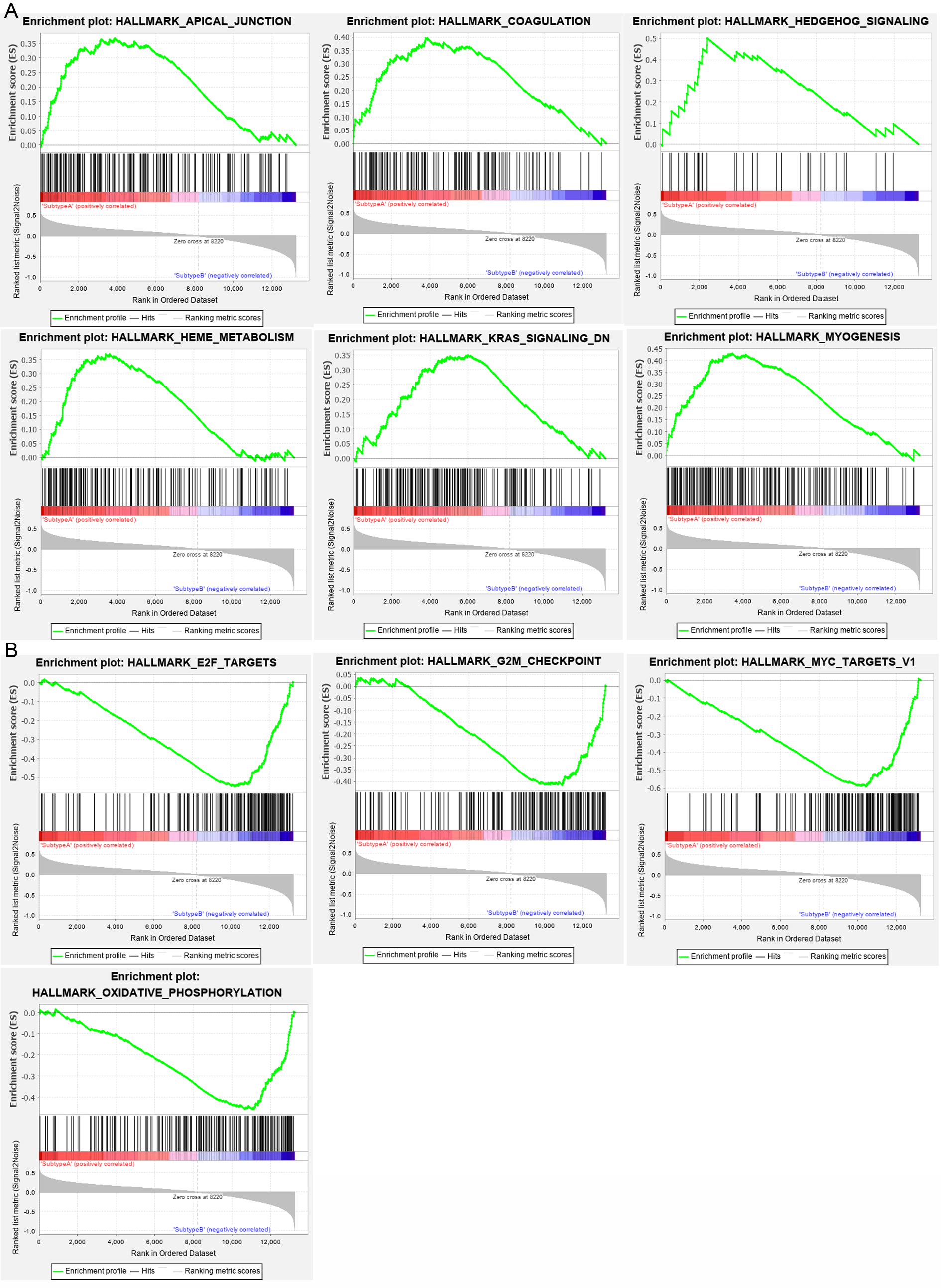

Supplement: Supplementary file 3 — Supplementary Figure S2. [file 41598_2024_53327_MOESM3_ESM.tif]
